# The impact of system interaction quality on learning outcomes in online virtual experiment teaching: the mediating role of extraneous cognitive load

**DOI:** 10.3389/fpsyg.2025.1739300

**Published:** 2026-01-21

**Authors:** Peng Yin, TingYu Sun

**Affiliations:** 1 Office of the President, Shandong Management University, Shandong, China; 2 Department of Labor Relations, Shandong Management University, Shandong, China

**Keywords:** communication quality, extraneous cognitive load, learning outcomes, online virtual experiment teaching, system interaction quality, user interface quality

## Abstract

This study investigates the effect of system interaction quality on learning outcomes in online virtual experiment teaching, with extraneous cognitive load serving as a mediating variable. Based on Cognitive Load Theory, a structural equation model was constructed to examine the relationships among user interface quality, communication quality, extraneous cognitive load, and learning outcomes. Using a cross-sectional questionnaire study, data collected from 610 valid samples were analyzed using SPSS 27.0 and AMOS 29.0. The results revealed that both user interface quality and communication quality significantly and positively predicted learning outcomes. Moreover, extraneous cognitive load partially mediated these relationships, indicating that high system interaction quality enhances learning outcomes not only directly but also indirectly by reducing unnecessary cognitive burdens. These findings extend the application of Cognitive Load Theory to virtual teaching contexts and provide empirical evidence for the “technology–cognition–learning” mechanism. Practically, the study offers actionable guidance for optimizing user interface design, improving communication performance, and enhancing instructional strategies to promote effective learning in online virtual experiment environments.

## 1 Introduction

Learning Outcome is a core indicator for measuring teaching effectiveness, a crucial reflection of educational quality, and a manifestation of the effectiveness of instructional system design and the implementation of teaching strategies (Hussey and Smith, 2008). As a multidimensional concept, learning outcome—from the perspective of educational objective taxonomy—encompass a progression from the memorization and comprehension of basic knowledge to application, analysis, synthesis, evaluation, and creation. They serve as fundamental criteria for verifying whether teaching activities have achieved their preset goals (Anderson and Krathwohl, 2001). Hussey and Smith (2008) defined learning outcome as verifiable learning achievements aligned with objectives, pointing out that learners' cognitive engagement and course quality are core influencing variables. Owing to the significance of learning outcome, existing studies have extensively explored this concept. Through a systematic review of studies conducted between 2009 and 2018, Martin et al. (2020) identified that teaching interaction, technical support, and learning design are key external factors affecting learning outcome. Additional studies have examined the predictive factors of learning outcomes in online courses, revealing that factors such as autonomous motivation, course design, and interaction with teachers and peers are associated with students' perceived learning outcomes (Wei et al., 2023).

Online virtual experiment teaching is a combination of online teaching and virtual technology (Li et al., 2018). Its application has become increasingly widespread in the global education field in recent years, and scholars have explored its learning outcome. In Virtual Experiment Teaching, learning outcome is specifically reflected in an in-depth understanding of experimental principles, proficiency in operating virtual instruments, and the ability to transfer problem-solving skills from simulated to real-world environments (Chang and Liu, 2025). Learning outcome not only embody the attainment of teaching objectives but also serve as a core basis for evaluating the quality of virtual experimental environment design (Nasrallah, 2014). Online virtual experiment teaching not only significantly reduces experimental costs and safety risks but also breaks the constraints of time, space, and resources, thereby enabling the widespread sharing of high-quality experimental teaching and enhancing both teaching efficiency and equity (Hamilton et al., 2021). However, the achievement of learning outcome in online virtual experiment teaching still faces numerous challenges. Many online virtual experiment teaching platforms are designed with a focus on the realization of technical functions, while neglecting learner-centered interaction experiences. Complex operation interfaces, unnatural feedback mechanisms, and unguided exploration processes increase learners' extraneous cognitive load, diverting their attention from understanding core concepts to coping with operational difficulties, thereby inhibiting deep learning (Mayer and Moreno, 2003). Therefore, enhancing the learning outcome of online virtual experiment teaching is crucial for guiding the development and iteration of online virtual experimental resources, as well as enhancing teaching quality and learning efficiency.

Although existing studies have discussed the impact of virtual technologies such as Augmented Reality (Dhar et al., 2021; Yusa et al., 2023) and Virtual Reality (Villena-Taranilla et al., 2022) on teaching effectiveness, most treat the technology itself as an independent variable rather than delving into which key features or mechanisms of the technology truly influence learning. Notably, the innovation of technical carriers does not equate to the success of online virtual experiment teaching. What matters more is whether teaching content, experimental resources, and other elements are effectively transmitted and transformed toward learning outcomes through technology. In online virtual experiment teaching, virtual experiment systems serve as the carrier of teaching. Learners can acquire knowledge through human-computer interaction or human-human interaction, both of which are conducted via virtual systems (Zhang et al., 2017). Against this backdrop, system interaction quality has become a common characteristic variable that transcends technical types and is inherent to all digital learning systems. Studies have indicated that system interaction is a key mediating link between learners and the virtual experimental environment. Its quality directly determines learners' information acquisition efficiency, operational experience, and depth of cognitive processing (Makransky and Mayer, 2022). Therefore, it exerts a significant impact on learning outcome. However, existing studies have not linked system interaction quality with learning outcome, nor have they conducted an in-depth analysis of the inherent influence mechanism between the two. Our study aims to investigate how system interaction quality affects learning outcome in online virtual experiment teaching and to uncover the underlying mechanisms, shifting the research focus from “technical effects” to “mechanism explanation.” This not only helps uncover the common mechanism of action behind different educational technologies but also provides a more universal theoretical basis for the design and optimization of educational technology systems.

To investigate the impact of system interaction quality on learning outcome and the underlying mechanism, this study incorporates the Cognitive Load Theory. First systematically proposed by Sweller (1988), the theory is based on the cognitive principle that human working memory has limited capacity. It categorizes cognitive load during learning into three types: Intrinsic Cognitive Load, Extraneous Cognitive Load, and Germane Cognitive Load (Sweller, 2011). Defects in the design of learning environments can trigger excessive extraneous cognitive load, which occupies limited working memory resources, thereby inhibiting knowledge encoding and transfer and ultimately reducing learning outcomes (Skulmowski and Xu, 2022). Additional studies have pointed out that optimizing the interaction design of learning environments can effectively reduce extraneous cognitive load. By alleviating cognitive burden, it creates conditions for deep learning, thereby significantly improving learning outcome (Klepsch and Seufert, 2020). In the context of online virtual experiment teaching, system interaction quality is directly related to learners' level of extraneous cognitive load, while the reasonable regulation of extraneous cognitive load affects the ultimate achievement of learning outcome. Therefore, this study attempts to further explore whether extraneous cognitive load plays a mediating role between system interaction quality and learning outcome in virtual experiment teaching, aiming to clarify the inherent correlation path among the three.

This study can reveal the mechanism of action between system interaction quality and learning outcome, helping educational technology designers and teachers to more effectively optimize system interaction design and improve learning performance. Based on this, the purpose of this study is to explore the impact of system interaction quality on learning outcome and verify the mediating role of extraneous cognitive load, thereby providing strong support for the optimization of educational informatization and intelligent learning environments. The main research questions of this study are:

1. Can system interaction quality significantly predict learning outcomes?

2. Can system interaction quality affect learning outcomes through the mediating role of extraneous cognitive load?

## 2 Theoretical background and research hypotheses

### 2.1 System interaction quality and learning outcomes

#### 2.1.1 Learning outcomes (LO)

Learning outcomes—often referred to as learning performance, learning efficiency, or academic achievement—comprehensively reflect the knowledge, skills, and attitudes that learners acquire over a period of study. They serve as key indicators for evaluating educational quality (Hussey and Smith, 2008). Previous research has classified learning outcomes into three categories: cognitive outcomes, behavioral outcomes, and affective outcomes (Wei et al., 2023). Cognitive outcomes refer to the knowledge and intellectual skills acquired by learners; behavioral outcomes pertain to the degree of engagement in learning activities; and affective outcomes relate to learners' satisfaction with and perceptions of the course (Wei et al., 2021). In the context of online virtual experiment teaching, learning outcomes manifest in two main ways. On the one hand, 3D modeling and interactive operations enable learners to intuitively grasp abstract concepts and complex principles. On the other hand, virtual experiments offer opportunities for repeated practice, reinforcing understanding and skill acquisition (Chang and Liu, 2025).

#### 2.1.2 System interaction quality (SIQ)

Interaction and communication among individuals constitute one of the fundamental characteristics of human society (Hier, 2005). In the information age, interactivity remains a key predictor of users' adoption of technologies and tools (Sun et al., 2024). System interaction quality was originally introduced as a component of the Information Systems Success Model, assessing users' interactive experience, fluency, and effectiveness during system use (Delone and McLean, 2003). In the educational context, virtual experiment teaching facilitates knowledge acquisition through the interactive use of computer simulations (Zhang et al., 2017). Notably, interaction among educational participants is a critical determinant of educational effectiveness. However, with the advancement of educational informatization and online teaching models, although learning convenience has improved, the interpersonal interaction characteristic of traditional classrooms has weakened (Xiao, 2017). Consequently, within the domain of modern educational technology, system interaction quality encompasses not only the quality of information exchange between learners and learning systems but also the extent of online communication among participants. It therefore comprises two dimensions: user interface quality and communication quality (Alhendawi and Baharudin, 2014). Among these, user interface quality (UIQ) primarily reflects the human–computer interaction experience between learners and the system, emphasizing factors such as intuitiveness and ease of use in interface design. Communication quality (CQ), on the other hand, represents the online interaction experience between learners and others (including instructors and peers), focusing on the timeliness and effectiveness of information exchange.

#### 2.1.3 System interaction quality and learning outcomes

The positive impact of system interaction quality has been extensively validated across multiple dimensions. Research indicates that the quality of a system's interactive design is positively correlated with perceived usefulness and employee performance (Lin, 2010). Furthermore, factors such as resource availability, feedback, and communication significantly enhance user motivation and satisfaction (Lawson-Body et al., 2010), which are, in turn, regarded as precursors to improved learning outcomes. In online learning environments, interactivity serves as a strong predictor of students' satisfaction with the learning system and their intention to continue using it (Cheng, 2020). High-quality interactivity not only optimizes users' emotional experiences and sense of immersion but also indirectly facilitates knowledge construction and learning performance by enhancing learners' cognitive engagement and social presence (Makransky and Mayer, 2022). Additionally, studies have confirmed that both dimensions of interaction quality—user interface quality and communication quality—positively influence system effectiveness and contribute to greater user satisfaction (Alhendawi and Baharudin, 2014).

Therefore, this study posits that system interaction quality in online virtual experiment teaching positively influences learning outcomes. Considering that the two dimensions of system interaction quality represent two primary types of interaction—human–computer interaction and human–human interaction—this study examines the effects of user interface quality and communication quality on learning outcomes separately. Based on this rationale, the following hypotheses are proposed to address Research Question 1:

H1: The user interface quality of the system in online virtual experiment teaching positively predicts learning outcomes.

H2: The communication quality of the system in online virtual experiment teaching positively predicts learning outcomes.

### 2.2 The mediating role of cognitive load theory and external cognitive load

#### 2.2.1 Cognitive Load Theory (CLT)

Cognitive Load Theory (CLT) was developed based on the structure of human cognition (Sweller, 2011), emphasizing the influence of the limited capacity of working memory (Cowan, 2014) on the learning process. When individuals encounter new information, it must first be processed through working memory—which has restricted capacity and duration—before being stored in long-term memory for future use (Chen et al., 2023). The theory highlights that the constraints of working memory are a key determinant of how effectively information can be presented (Haryana et al., 2022). Cognitive load refers to the amount of working memory resources expended by an individual when processing information during a learning task (Sweller, 2011).

One of the core concepts in CLT is element interactivity, which describes situations in which information consists of multiple interacting elements that must be processed simultaneously (Sweller, 2024). Based on the effects of element interactivity on working and long-term memory, cognitive load was originally categorized into three types: intrinsic, extraneous, and germane cognitive load. Intrinsic cognitive load refers to the working memory demands imposed by the inherent complexity of the learning content. Extraneous cognitive load represents the additional demands arising from the way information is presented or organized. Germane cognitive load, meanwhile, refers to the working memory resources required for learning itself (Sweller et al., 1998). Over the past two decades, considerable debate has emerged concerning the nature and validity of germane cognitive load (Skulmowski and Xu, 2022). In response, Sweller et al. (2019) proposed a revised version of CLT, replacing “germane cognitive load” with the term “germane processing” and excluding it as a constituent component of total cognitive load.

#### 2.2.2 The mediating role of external cognitive load (ECL)

Extraneous cognitive load pertains to the way information is presented and processed (Sweller et al., 1998). Hollender et al. (2010) defined it as the combined cognitive load generated by instructional design and software usability. Specifically, it refers to the cognitive burden that is unrelated to learning objectives and arises from poorly designed instruction or inappropriate methods of information presentation (Sweller, 2011). In online virtual teaching environments, extraneous cognitive load is influenced not only by instructions and explanations but also by interactive technologies and the broader learning environment. Accordingly, in online virtual experiment teaching, extraneous cognitive load can be classified into three dimensions: extraneous load instruction, extraneous load interaction, and extraneous load environment (Andersen and Makransky, 2021).

Factors such as the format of task presentation or the learning environment can serve as sources of extraneous cognitive load (Schnotz and Kürschner, 2007). A key feature of virtual experiment teaching is its reliance on system interactions to deliver information (Haryana et al., 2022). In this context, the quality of system interaction may act as a presentation format for learning tasks, potentially inducing extraneous cognitive load in learners. Learners' perception and processing of new information or knowledge vary depending on the medium through which the information is transmitted (Daghestani et al., 2012). Previous studies have shown that when a system interface is poorly designed, learners must expend additional cognitive resources to understand the interface logic, locate relevant information, or process redundant content, thereby increasing extraneous cognitive load (Makransky and Mayer, 2022). Similarly, communication barriers—such as ineffective interaction or delayed feedback during the learning process—can also heighten extraneous cognitive load (Costley and Fanguy, 2021). Therefore, both the user interface quality and communication quality of a system may increase extraneous cognitive load. Since extraneous cognitive load does not facilitate knowledge construction but instead consumes learners' limited cognitive resources, it can hinder deep processing and comprehension (Skulmowski and Xu, 2022). Prior research has confirmed that excessive extraneous load reduces instructional effectiveness; thus, instructional design should aim to minimize or eliminate this type of cognitive load (Sweller, 2023). Consequently, this study posits that system interaction quality in online virtual experiment teaching indirectly influences learning outcomes through extraneous cognitive load. Based on this rationale, the following hypotheses are proposed to address Research Question 2:

H3: Extraneous cognitive load mediates the effect of communication quality on learning outcomes.

H4: Extraneous cognitive load mediates the effect of user interface quality on learning outcomes.

**Figure 1:**
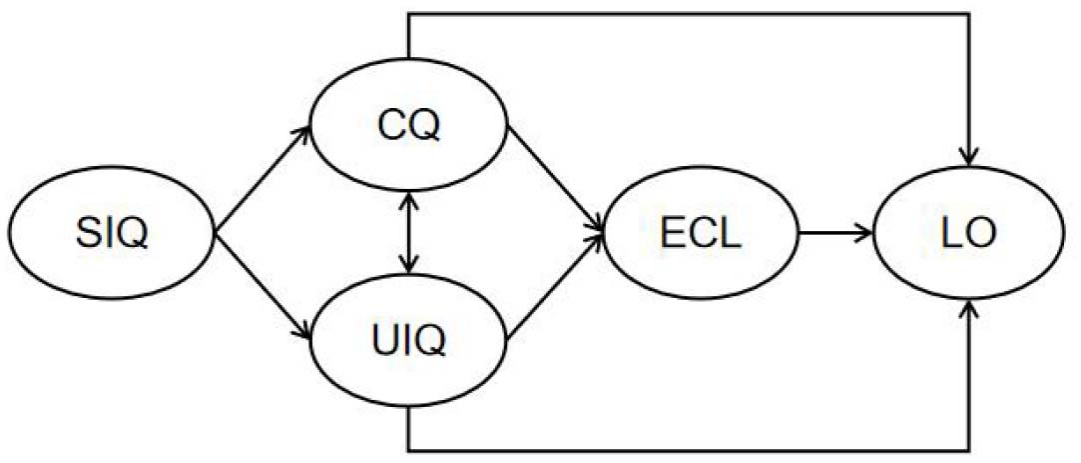
Research model. SIQ, System Interaction Quality; CQ, Communication Quality; UIQ, User Interface Quality; ECL, Extraneous Cognitive Load; LO, Learning Outcome.

Based on the theoretical background and the hypotheses outlined above, this study develops a hypothesized model illustrating the relationships among system interaction quality, extraneous cognitive load, and learning outcomes, as shown in Figure 1.

## 3 Methods

### 3.1 Participants and procedure

This study adopted a convenience sampling method. In October 2025, an online survey was conducted using the Questionnaire Star platform as the data collection tool. The participants were students from higher education institutions in Shandong Province who had participated in online virtual experiment teaching during the current semester. After reading the informed consent form, participants could voluntarily choose whether to access the survey link and complete the questionnaire. A total of 678 questionnaires were collected during the survey period. To ensure the quality of the data, responses with logical inconsistencies, patterned answering, or excessively short completion times were excluded. Ultimately, 610 valid questionnaires remained, yielding an effective response rate of 89.9%. All participants were current university students, with a mean age of 20.1 years. Among them, 321 were from undergraduate institutions and 289 from vocational colleges, accounting for 52.6% and 47.4%, respectively. Regarding academic disciplines, 189 students (31%) were from natural sciences, 246 (40.3%) from engineering sciences, 124 (20.3%) from medical sciences, and 51 (8.4%) from humanities and social sciences. In terms of gender, there were 355 male students (58.2%) and 255 female students (41.8%). Details are shown in Table 1.

**Table 1:** General demographic characteristics (*n* = 610).

**Descriptor**	**Category**	***n***	**%**	**Descriptor**	**Category**	***n***	**%**
Gender	Male	355	58.2%	Discipline	Natural science	189	31%
	Female	255	41.8%		Engineering science	246	40.3%
Type of Institution	Undergraduate universities	321	52.6%		Medical science	124	20.3%
	Vocational colleges	289	47.4%		Humanities and social sciences	51	8.4%

### 3.2 Measures

#### 3.2.1 Learning Outcome (LO)

This study employed the Perceived Learning outcome Scale developed by DiLoreto et al. (2022) to measure learning outcome. The scale consists of four items (e.g., “The learning tasks deepened my understanding of the course content”). A six-point Likert scale (1 = “strongly disagree,” 6 = “strongly agree”) was used, and higher scores indicate higher levels of perceived learning outcome. The Cronbach's α coefficient for this scale was 0.793. Confirmatory factor analysis (CFA) showed that the model fit indices (χ^2^/df = 2.101, RMSEA = 0.043, CFI = 0.997, GFI = 0.997, NFI = 0.994) met the standard criteria for good model fit, indicating that the scale demonstrated satisfactory structural validity.

#### 3.2.2 System Interaction Quality (SIQ)

The System Interaction Quality was measured with the 10-item scale developed by Alhendawi and Baharudin (2014). It includes two dimensions: Communication Quality (UIQ, 5 items, e.g., “The system provides discussion boards”) and User Interface Quality (CQ, 5 items, e.g., “The design of system services facilitates access”). All items were rated on a 7-point Likert scale (1 = “strongly disagree”, 7 = “strongly agree”), and higher scores indicating better system interaction quality. The scale demonstrated a Cronbach's α coefficient of 0.832. Confirmatory factor analysis revealed that the structural fit indices of the scale (χ^2^/df = 1.444, RMSEA = 0.027, CFI = 0.993, GFI = 0.986, NFI = 0.979) met the recommended criteria, indicating good structural validity of the scale.

#### 3.2.3 Extraneous Cognitive Load (ECL)

The Extraneous Cognitive Load was measured with the 11-item scale developed Andersen and Makransky (2021). It includes three dimensions: extraneous load instruction (3 items, e.g., “The instructions and/or explanations used during the simulation were very unclear”), extraneous load interaction (4 items, e.g., “The interactive technology used in the simulation made learning more difficult”), and extraneous load environment (4 items, e.g., “Various elements in the virtual environment made learning very confusing”). All items were rated on a 9-point Likert scale (1 = “strongly disagree,” 7 = “strongly agree”), and higher scores indicating a higher level of extraneous cognitive load. The scale demonstrated a Cronbach's α coefficient of 0.944. Confirmatory factor analysis revealed that the structural fit indices of the scale (χ^2^/df = 1.363, RMSEA = 0.024, CFI = 0.997, GFI = 0.984, NFI = 0.988) met the recommended criteria, indicating good structural validity of the scale.

#### 3.2.4 Analytical strategy

This study employed SPSS 27.0 to conduct descriptive analysis, correlation analysis, and common method bias tests on the data. AMOS 29.0 was used to perform confirmatory factor analysis and establish a structural equation model to investigate the impact of system interaction quality on learning outcome in online virtual experiment teaching. The Bootstrap method was applied to examine the mediating effect of extraneous cognitive load between these two variables.

## 4 Results

### 4.1 Common method biases

To minimize common method bias in the data, we first implemented controls in the survey procedure, such as ensuring anonymity and confidentiality, including reverse-scored items, avoiding ambiguous or leading language, and using different numbers of Likert scale points. Secondly, Harman's single-factor test was employed to assess common method bias in the collected data. An unrotated exploratory factor analysis of all items yielded a KMO value of 0.949 (*p* < 0.001), indicating suitability for factor analysis. The first factor accounted for 36.286% of the variance, which is below the critical threshold of 40%, suggesting no significant common method bias (Podsakoff et al., 2003).

### 4.2 Descriptive statistics and correlations

Descriptive statistics and Pearson correlation analysis were conducted on the data, and the results are presented in Table 2. Significant correlations (*p* < 0.01) were observed among the four core variables: user interface quality showed significant positive correlations with communication quality (*r* = 0.300) and learning outcome (r = 0.399), and a significant negative correlation with extraneous cognitive load (*r* = −0.389). Communication quality was significantly positively correlated with learning outcome (*r* = 0.415) and significantly negatively correlated with extraneous cognitive load (*r* = −0.317). Extraneous cognitive load demonstrated a significant negative correlation with learning outcome (*r* = −0.393). The correlations among the variables provide preliminary support for subsequent hypothesis testing.

**Table 2:** Descriptive statistics, correlations and AVE values.

**Variable**	**M**	**SD**	**CQ**	**UIQ**	**ECL**	**LO**
CQ	3.995	1.602	**0.721**			
UIQ	3.999	1.582	0.300**	**0.717**		
ECL	5.545	1.950	−0.389**	−0.317**	**0.779**	
LO	3.687	1.343	0.399**	0.415**	−0.393**	**0.702**

** *p* < 0.01. Bold value indicates diagonal values are square roots of AVE values. CQ: Communication Quality; UIQ: User Interface Quality; ECL: Extraneous Cognitive Load; LO: Learning Outcome.

### 4.3 Convergent and discriminant validity tests

The tests revealed that all standardized factor loadings (β) for the measurement items exceeded 0.6 (Costello and Osborne, 2005), with values ranging from 0.682 to 0.769 for user interface quality, 0.672 to 0.759 for communication quality, 0.915 to 0.934 for extraneous cognitive load, and 0.653 to 0.739 for learning outcome. All loadings were statistically significant (*p* < 0.001), indicating strong associations and representativeness between the measurement items and their respective constructs. The calculation of average variance extracted (AVE) and composite reliability (CR) showed that only the AVE for learning outcome was slightly below 0.5, though values above 0.4 are generally considered acceptable (Verhoef et al., 2002). All constructs demonstrated CR values exceeding 0.7, confirming high internal consistency and convergent validity (Fornell and Larcker, 1981). Detailed results are presented in Table 3.

**Table 3:** Results of convergent validity.

**Variables**	**Measurement item**	**B**	**β**	**S.E**.	**C.R**.	***p***	**CR**	**AVE**
Communication quality	CQ1	1.000	0.682	/	/	/	0.520	0.884
	CQ2	1.052	0.713	0.069	15.165	<0.001		
	CQ3	1.107	0.769	0.069	16.119	<0.001		
	CQ4	1.099	0.738	0.070	15.61	<0.001		
	CQ5	1.020	0.701	0.068	14.96	<0.001		
User interface quality	UIQ1	1.000	0.712	/	/	/	0.513	0.840
	UIQ2	1.077	0.747	0.027	35.877	<0.001		
	UIQ3	1.115	0.759	0.066	16.328	<0.001		
	UIQ4	1.033	0.689	0.067	16.553	<0.001		
	UIQ5	0.992	0.672	0.068	15.21	<0.001		
Extraneous cognitive load	ECL1	1.000	0.915	/	/	/	0.859	0.948
	ECL2	1.000	0.931	0.025	39.206	<0.001		
	ECL3	1.012	0.934	0.026	39.552	<0.001		
Learning outcome	LO1	1.000	0.653	/	/	/	0.492	0.795
	LO2	1.092	0.739	0.077	14.266	<0.001		
	LO3	0.996	0.681	0.074	13.454	<0.001		
	LO4	1.104	0.731	0.078	14.137	<0.001		

CQ, Communication Quality; UIQ, User Interface Quality; ECL, Extraneous Cognitive Load; LO, Learning Outcome.

Discriminant validity among the constructs was tested, with the results shown in Table 2. The values on the diagonal represent the square roots of the AVE for each variable, while the off-diagonal values indicate the correlation coefficients between variables. The results demonstrate that all correlation coefficients between constructs were significantly lower than the square roots of the AVE in their respective rows and columns. This indicates good discriminant validity (Fornell and Larcker, 1981).

### 4.4 Hypothesis testing and path analysis

**Figure 2:**
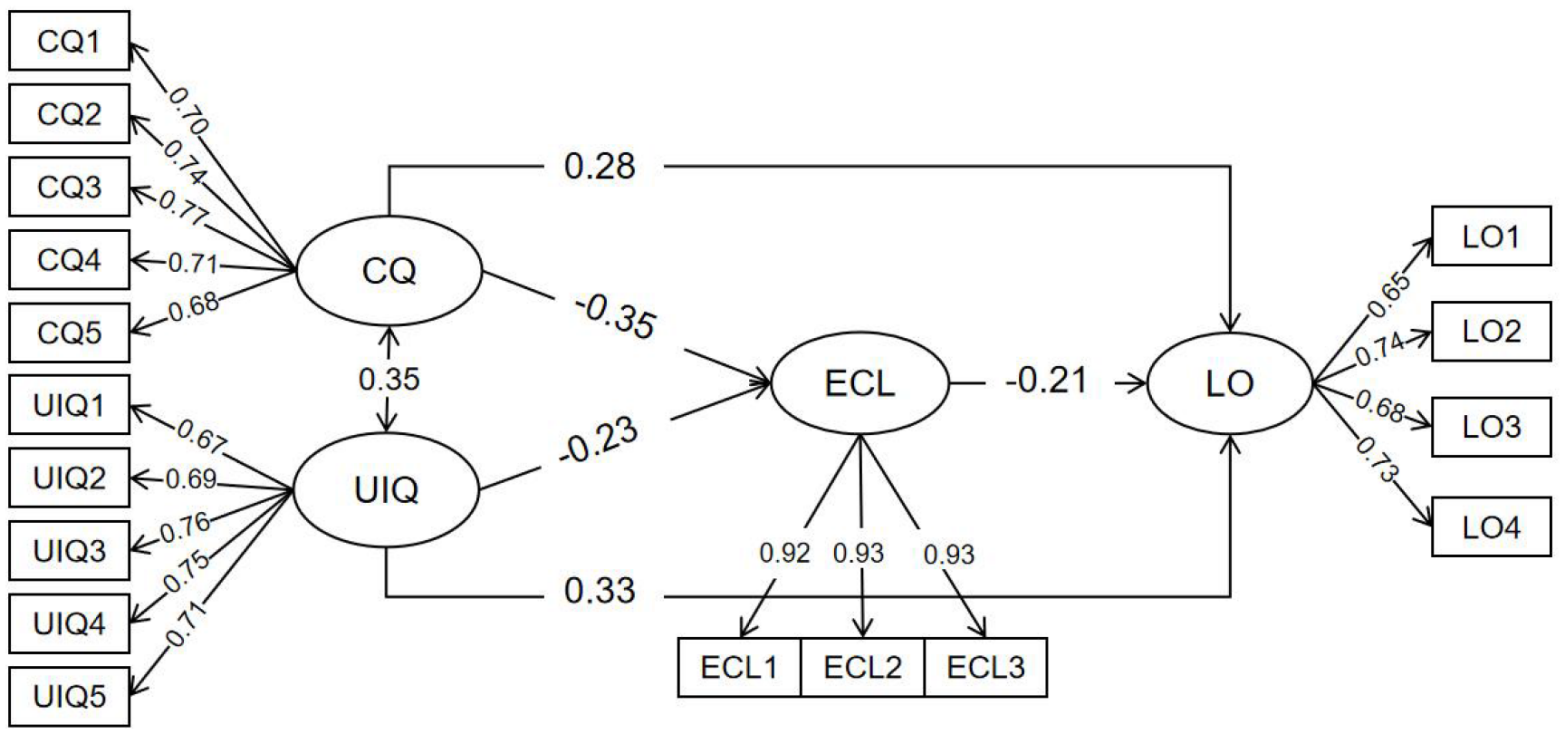
Structural equation model. SIQ, System Interaction Quality; CQ, Communication Quality; UIQ, User Interface Quality; ECL, Extraneous Cognitive Load; LO, Learning Outcome.

To test the aforementioned hypotheses, a structural equation model was constructed with communication quality and user interface quality as independent variables, learning outcome as the dependent variable, and extraneous cognitive load as the mediating variable, while controlling for the demographic variables of gender and major. The model structure is shown in Figure 2. The results of the confirmatory factor analysis for the model were as follows: χ^2^/df = 1.165, RMSEA = 0.016, NFI = 0.975, CFI = 0.996, TLI = 0.996, indicating a good model fit (Kline, 2023).

To further examine the role of the mediating variable, the bias-corrected percentile bootstrap method was employed to test the significance of the mediating effects. Using a 95% confidence interval, the data were resampled 5,000 times. Significance was indicated if the confidence interval did not include 0. The test results are presented in Table 4.

**Table 4:** Bootstrap mediation effect.

**Independent variable**	**Paths**		**Effects**	***P***	**95% CI**		**Relative mediation effect (%)**
					**Lower**	**Upper**	
CQ	CQ → ECL		0.353	< 0.001	−0.432	−0.270	/
	ECL → LO		0.212	< 0.001	−0.299	−0.117	/
	CQ → LO	Direct path: CQ → LO	0.276	< 0.001	0.178	0.371	78.7%
		Indirect path: CQ → ECL → LO	0.075	< 0.001	0.042	0.115	21.3%
		Total effect	0.351	< 0.001	0.258	0.439	100
UIQ	UIQ → ECL		0.231	< 0.001	−0.315	−0.139	/
	ECL → LO		0.212	< 0.001	−0.299	−0.117	/
	UIQ → LO	Direct path: UIQ → LO	0.331	< 0.001	0.229	0.420	87.1%
		Indirect path: UIQ → ECL → LO	0.049	< 0.001	0.025	0.082	12.9%
		Total effect	0.627	< 0.001	0.577	0.722	100

CQ, Communication Quality; UIQ, User Interface Quality; ECL, Extraneous Cognitive Load; LO, Learning Outcome.

Regarding the total effect of system interaction quality on learning outcome, both communication quality (β = 0.351, p <0.001) and user interface quality (β = 0.379, *p* < 0.001) had significant direct effects on learning outcome when the mediating variable was not included, supporting Hypotheses H1 and H2.

After introducing extraneous cognitive load as a mediating variable, the effect of communication quality on extraneous cognitive load was −0.353 (*p* <0.001), with a 95% confidence interval (CI) of [−0.432, −0.270], as the CI did not include 0, indicating that communication quality significantly reduces extraneous cognitive load. The effect of extraneous cognitive load on learning outcome was −0.212 (*p* < 0.001), with a 95% CI of [−0.299, −0.117], indicating that extraneous cognitive load significantly decreases learning outcome. When extraneous cognitive load was included as a mediator, the indirect path from communication quality → extraneous cognitive load → learning outcome had an effect of 0.075 (*p* < 0.001), with a 95% CI of [0.042, 0.115], as the CI did not include 0. Furthermore, after including the mediator, the direct effect of communication quality on learning outcome remained significant, with an effect of 0.276 (*p* <0.001) and a 95% CI of [0.178, 0.371]. These results indicate that extraneous cognitive load plays a significant partial mediating role between communication quality and learning outcome. The proportions of the direct and indirect effects were 78.7% and 21.3%, respectively, supporting Hypothesis H3.

After introducing extraneous cognitive load as a mediating variable, the effect of user interface quality on extraneous cognitive load was −0.231 (*p* <0.001), with a 95% confidence interval (CI) of [−0.315, −0.139], as the CI did not include 0, indicating that user interface quality significantly reduces extraneous cognitive load. The indirect path from user interface quality → extraneous cognitive load → learning outcome had an effect of 0.049 (*p* < 0.001), with a 95% CI of [0.025, 0.082], as the CI did not include 0. Furthermore, after including the mediator, the direct effect of user interface quality on learning outcome remained significant, with an effect of 0.331 (*p* <0.001) and a 95% CI of [0.229, 0.420]. These results indicate that extraneous cognitive load plays a significant partial mediating role between user interface quality and learning outcome. The proportions of the direct and indirect effects were 87.1% and 12.9%, respectively, supporting Hypothesis H4.

## 5 Discussion

This study aimed to investigate how system interaction quality affects learning outcomes in online virtual experiment teaching, with extraneous cognitive load functioning as a mediating mechanism. Grounded in Cognitive Load Theory, the study focused on two key aspects of system interaction quality—user interface quality and communication quality—and examined their respective impacts on learners' performance. The following discussion interprets the empirical findings and elaborates on their theoretical and practical implications within the context of online virtual experiment learning environments.

First, both communication quality and user interface quality, as components of system interaction quality, significantly and positively predict learning outcomes.

From the perspective of user interface quality, this result aligns with previous research suggesting that the ease of use of digital tools reduces technological anxiety and enhances teaching quality (Ndirangu and Udoto, 2011; Stefanovic and Klochkova, 2021); it also supports the view that the adaptability of digital technology promotes teaching effectiveness (Fütterer et al., 2023). User interface quality not only affects the convenience of learning operations but also influences learners' emotional experiences and self-efficacy (Bollini, 2017). Moreover, the aesthetic appeal and logical design of the interface can enhance learners' trust and sense of control over the system, thereby stimulating their learning motivation and independent exploration behaviors. This, in turn, leads to higher levels of concentration and engagement during virtual experiments (Hui and See, 2015).

From the perspective of communication quality, previous research has identified interaction as crucial for learning outcome and satisfaction (Tsang et al., 2021; Zhang et al., 2017). Our results confirm that interaction remains equally important in online virtual experiment teaching, with communication quality serving as the foundation for effective interaction in digital environments. Considering the unique requirements of online virtual experiment teaching scenarios, learners' ability to deepen knowledge comprehension and master experimental skills depends on reliable communication quality. This quality facilitates real-time teacher-student Q&A and peer collaborative feedback, enabling timely and effective interactive support (Quadir et al., 2022). If the system's communication quality is compromised, it directly undermines the online interaction experience. Delays in resolving learning queries may trigger negative emotions such as frustration and discouragement among learners, ultimately reducing their engagement and concentration (Rubio-Tamayo et al., 2017; Shang et al., 2022).

Unlike previous studies, this study does not focus on a single technological platform but instead adopts a structural perspective on system interaction quality, allowing a comprehensive examination of the roles of two distinct interaction dimensions. This multidimensional analysis addresses the limitation of prior studies that treated “interaction” as a unitary concept, providing a clearer theoretical delineation of system interaction quality. The results demonstrate that both interface quality at the human-computer interaction level and communication quality at the interpersonal level significantly enhance learning outcome.

Second, system interaction quality enhances learning outcome by reducing extraneous cognitive load.

This study further reveals that both user interface quality and communication quality not only directly improve learning outcome but also indirectly improve them by reducing extraneous cognitive load. This finding elucidates the psychological mechanism through which interaction quality influences learning outcome from a cognitive processing perspective, aligning with the core premise of cognitive load theory (Sweller, 2024; Sweller et al., 1998): learning system design should minimize cognitive burdens unrelated to learning objectives, enabling learners to allocate their limited cognitive resources to core learning tasks. Given learners' finite cognitive resources, extraneous cognitive load acts as an “inefficient cognitive consumption.” Excessive extraneous cognitive load can divert resources away from essential learning tasks, leading to diminished learning outcome (Skulmowski and Xu, 2022). Cognitive load theory posits that extraneous cognitive load can be regulated through environmental design. When extraneous cognitive load is effectively managed via system interaction quality, learners' cognitive resources are optimally allocated, facilitating deeper learning and thereby improving learning outcome (Klepsch and Seufert, 2020). This further validates the mediating role of extraneous cognitive load as a “regulator of cognitive resource allocation.”

The results of this study address the research gap concerning the relationship between system interaction quality, extraneous cognitive load, and learning outcome. It shifts the understanding of system interaction quality from a technical perspective to a cognitive dimension, while simultaneously validating the mediating pathway from system interaction quality through extraneous cognitive load to learning outcome. This not only extends the applicability of cognitive load theory in virtual teaching contexts but also highlights the critical role of technical experience variables in cognitive processing. These insights provide a theoretical foundation for future research on the technology-cognition-learning mechanism.

Third, unlike previous studies that focused on specific technology types, this research adopts a system interaction quality perspective to explore universal mechanisms across different technologies. The main contributions of this study are as follows:

At the theoretical level, this study reveals the psychological mechanism through which system interaction quality influences learning outcome via extraneous cognitive load, thus extending the application of cognitive load theory to online virtual teaching. At the empirical level, this study categorizes system interaction quality into two dimensions—user interface quality and communication quality—and constructs and validates a model linking system interaction quality, extraneous cognitive load, and learning outcome. This uncovers the distinct pathways through which different interaction dimensions operate, providing a new measurement and analytical framework for related research.

At the practical level, the findings offer actionable directions for optimizing interface design, communication performance, and instructional activities in online virtual teaching systems, thereby enhancing online learning experiences and improving learning outcomes. Firstly, developers should prioritize reducing extraneous cognitive load by optimizing interface information architecture, minimizing redundant animations and complex operations, and incorporating intelligent design features (such as automatic prompts and task progress guidance) to reduce learners' cognitive and operational load (Yan et al., 2021). For instance, the learning management system (LMS) developed by Suryani et al. (2024) incorporates an adaptive interface design that dynamically captures learners' cognitive states—including prior knowledge, working memory capacity, and perceived task complexity—and uses this data to personalize interface elements in real time. Secondly, the performance and interaction quality of communication modules should be enhanced to ensure smooth information flow and immediate feedback during the learning process, thereby preventing cognitive disruptions caused by delays, lags, or misunderstandings (Skowronek and Raake, 2015). As an example, Kerimbayev et al. (2020) constructed a multi-layered, multimodal interactive virtual learning environment based on the Moodle platform, integrating teacher–student, student–student, and human–computer interactions, while effectively combining synchronous and asynchronous communication modes. At the teacher level, it is essential to appropriately pace online interactions and avoid excessive communication and information overload, to help students maintain focus on core learning content (Xie et al., 2023).

## 6 Conclusion

This study, grounded in Cognitive Load Theory, constructed a structural equation model with two sub dimensions of system interaction quality—communication quality and user interface quality—as independent variables, extraneous cognitive load as the mediating variable, and learning outcomes as the dependent variable. The results demonstrate that system interaction quality plays a crucial role in shaping learning outcomes in online virtual experiment teaching, with both user interface quality and communication quality significantly enhancing learning performance. Furthermore, extraneous cognitive load partially mediates the relationship between system interaction quality and learning outcomes. This research extends the application of Cognitive Load Theory to the field of online virtual teaching and provides practical guidance for improving interface design, optimizing communication, and designing instructional activities in online virtual teaching systems.

## 7 Limitations and future directions

Despite its valuable contributions, this study has several limitations.

First, the data in this study were collected via self-reported questionnaires. Although certain procedural controls were implemented, common method bias and the influence of subjective perceptions cannot be entirely ruled out. Future research could incorporate objective measures such as test performance or physiological data. For instance, physiological measures (e.g., eye-tracking, heart rate variability) or experimental task performance (e.g., immediate test scores) could be used to cross-validate self-reported responses, while multi-source data triangulation (e.g., combining teacher evaluations, student self-reports, and system logs) could enhance construct validity.

Second, the cross-sectional design restricts causal interpretation. To enhance causal inference, future research could adopt the following designs: a longitudinal approach, measuring system interaction quality, extraneous cognitive load, and learning outcomes at multiple time points to analyze temporal relationships through cross-lagged panel modeling; and an experimental approach, in which students are randomly assigned to virtual learning environments with either high or low interface/communication quality while controlling for other variables. This would allow direct observation of changes in extraneous cognitive load and learning outcomes, thereby helping to establish a causal chain.

Third, this study focused on system-level and cognitive-level variables and did not include individual learner characteristics as predictors in the model. The absence of these individual factors implies that potential confounding effects cannot be fully discounted. Future studies could incorporate learner characteristics—such as prior knowledge, technical proficiency, or working memory capacity—to provide a more comprehensive understanding of the interplay between system design and learner differences in virtual learning environments.

## Data Availability

The raw data supporting the conclusions of this article will be made available by the authors, without undue reservation.
